# P206: Stenotrophomonas maltophilia bacteraemia: analysis of 33 episodes occurred in the ICU at the University Hospital in Sousse-Tunisia

**DOI:** 10.1186/2047-2994-2-S1-P206

**Published:** 2013-06-20

**Authors:** O Bouallègue, N Jaidane, H Said laatiri, W Naija, S Khefecha Aissa, N Boujaafar, L Dhidah

**Affiliations:** 1 Microbiology Laboratory, Department of Hygiene and Service reanimation, Hospital of Sahloul, Sousse, Tunisia

## Introduction

*Stenotrophomonas maltophilia* is a gram negative bacillus that has emerged as an opportunistic pathogen associated with high morbidity and mortality rates.

## Objectives

The aim of this study is to describe the characteristics of bactaeremia due to this strain, their outcome, the antibiotic sensitivity patterns of isolates.

## Results

In our study, 93% of 33 episodes were nosocomial. There were 22 deaths (71%) 15±12 days after the bactaeremia. 17/31 of patients were exposed to broad-spectrum antibiotic specifically imipinem (IMP) before their positive culture. Among cases, 23 (74%) patient had mechanical ventilation and 29 (93·5%) had central venous catheterization. Antibiotic susceptibility testing revealed that isolates were most sensitive to Ciprofloxacin (CIP) (84%), Trimethoprim-sulfamethoxazole (SXT) (71%) and to Colistin (CS) (58%). Twenty three percent (23%) episodes were polymicrobial. A probable portal of entry was identified in 27·3% of bacteraemic episodes and 57% were catheter-related. Fifty eight percent (58%) of the episodes were treated with monotherapy specifically CIP (35%). Our results were similar to those described by others in the last 20 years. These studies have been mostly retrospective.

## Conclusion

Prevention of *S. maltophilia* infection relies on the cornerstones of modern infection control, such as higher emphasis on control of antimicrobial consumption and consideration of environmental reservoirs.

## Disclosure of interest

None declared

